# An oligosaccharyltransferase from *Leishmania major* increases the N‐glycan occupancy on recombinant glycoproteins produced in *Nicotiana benthamiana*

**DOI:** 10.1111/pbi.12906

**Published:** 2018-03-25

**Authors:** Alexandra Castilho, Gernot Beihammer, Christina Pfeiffer, Kathrin Göritzer, Laura Montero‐Morales, Ulrike Vavra, Daniel Maresch, Clemens Grünwald‐Gruber, Friedrich Altmann, Herta Steinkellner, Richard Strasser

**Affiliations:** ^1^ Department of Applied Genetics and Cell Biology University of Natural Resources and Life Sciences Vienna Austria; ^2^ Department of Chemistry University of Natural Resources and Life Sciences Vienna Austria

**Keywords:** glyco‐engineering, N‐glycosylation, *Nicotiana benthamiana*, oligosaccharyltransferase, plant‐made pharmaceuticals

## Abstract

N‐glycosylation is critical for recombinant glycoprotein production as it influences the heterogeneity of products and affects their biological function. In most eukaryotes, the oligosaccharyltransferase is the central‐protein complex facilitating the N‐glycosylation of proteins in the lumen of the endoplasmic reticulum (ER). Not all potential N‐glycosylation sites are recognized *in vivo* and the site occupancy can vary in different expression systems, resulting in underglycosylation of recombinant glycoproteins. To overcome this limitation in plants, we expressed LmSTT3D, a single‐subunit oligosaccharyltransferase from the protozoan *Leishmania major* transiently in *Nicotiana benthamiana,* a well‐established production platform for recombinant proteins. A fluorescent protein‐tagged LmSTT3D variant was predominately found in the ER and co‐located with plant oligosaccharyltransferase subunits. Co‐expression of LmSTT3D with immunoglobulins and other recombinant human glycoproteins resulted in a substantially increased N‐glycosylation site occupancy on all N‐glycosylation sites except those that were already more than 90% occupied. Our results show that the heterologous expression of LmSTT3D is a versatile tool to increase N‐glycosylation efficiency in plants.

## Introduction

Asparagine (N)‐linked glycosylation is a major co‐ and post‐translational modification of proteins entering the secretory pathway. Many recombinant biopharmaceuticals for therapeutic use in humans are N‐glycosylated, and distinct N‐glycan structures play crucial roles for their *in vivo* efficacy (Jefferis, 2009; Zacchi and Schulz, 2016). Yet, the extent of N‐glycan attachment to a distinct glycosylation site may vary greatly giving rise to the formation of incompletely glycosylated proteins with potentially unwanted characteristics. N‐glycans are important for protein folding and protein stability and specifically modulate protein–protein interactions. For erythropoietin (EPO), it has been shown that the *in vivo* biological activity correlates with the number of N‐linked glycans (Elliott *et al*., 2004) and nonglycosylated monoclonal antibodies display reduced or complete loss of immune receptor binding (Nose and Wigzell, 1983; Walker *et al*., 1989).

In all eukaryotes, a hallmark of N‐glycosylation is the *en bloc* transfer of a common preassembled oligosaccharide (Glc_3_Man_9_GlcNAc_2_) from the lipid carrier dolichol pyrophosphate to selected asparagine residues in the sequence Asn‐X‐Ser/Thr (X any amino acid except proline) of nascent polypeptides (Aebi, 2013; Zielinska *et al*., 2010). The transfer of the oligosaccharide takes place in the lumen of the ER and is catalysed by the oligosaccharyltransferase (OST) complex. In yeast and mammals, OST is a multimeric membrane‐bound protein complex (Kelleher and Gilmore, 2006) consisting of one catalytically active subunit (STT3) and several different noncatalytic subunits that contribute to N‐glycosylation by regulation of the substrate specificity, stability or assembly of the complex (Knauer and Lehle, 1999; Mohorko *et al*., 2011; Yan and Lennarz, 2002). The organization of the OST complex is more complex in metazoans than in yeast, and different subunit compositions have been described (Mohorko *et al*., 2011; Roboti and High, 2012; Shibatani *et al*., 2005). Mammals harbour two different catalytic STT3 isoforms (STT3A and STT3B) that are present in distinct OST complexes (Ruiz‐Canada *et al*., 2009; Shrimal *et al*., 2013, 2015). The STT3A/STT3B‐containing complexes have overlapping and isoform specific functions and differ in their catalytic activity and acceptor substrate selectivity. While STT3A is predominately involved in co‐translational glycosylation, STT3B displays a preference for post‐translational glycosylation. By contrast, some unicellular parasites like *Leishmania major* or *Trypanosoma brucei* have several STT3 copies, but lack other noncatalytic subunits of the yeast or mammalian OST complex (Kelleher and Gilmore, 2006; Samuelson *et al*., 2005). These single‐subunit OST enzymes display distinct protein acceptor and oligosaccharide donor specificities (Izquierdo *et al*., 2009; Nasab *et al*., 2008).

N‐glycosylation in plants requires a similar heteromeric OST complex, which is still poorly described (Strasser, 2016). *Arabidopsis thaliana* has two catalytic subunits, termed STT3A and STT3B (Koiwa *et al*., 2003). STT3A‐deficient plants are viable, but display a protein underglycosylation defect that disturbs the biogenesis of different proteins including the heavily glycosylated pattern recognition receptor EF‐TU RECEPTOR (EFR), the endo‐β1,4‐glucanase KORRIGAN1 (KOR1/RSW2) (Kang *et al*., 2008) or the myrosinase TGG1 (Koiwa *et al*., 2003; Nekrasov *et al*., 2009; Saijo *et al*., 2009). Moreover, the *A. thaliana stt3a stt3b* double knockout mutant is gametophytic lethal (Koiwa *et al*., 2003) highlighting the importance of the catalytic OST subunits for protein N‐glycosylation in plants.

Plants are increasingly used as production hosts for recombinant human glycoproteins intended for therapeutic use. The majority of the approved recombinant biopharmaceuticals like monoclonal antibodies are glycoproteins and N‐glycosylation modulates, for example, the IgG function by affecting the binding affinity to receptors on immune cells. In recent years, enormous efforts have been made to engineer plant‐based expression hosts for the production of glycoproteins with targeted glycan profiles (Dicker *et al*., 2016; Hanania *et al*., 2017; Kallolimath *et al*., 2016; Li *et al*., 2016; Limkul *et al*., 2016; Loos *et al*., 2014; Mercx *et al*., 2017; Strasser *et al*., 2014). These advances reduced the variation of N‐glycan structures on a given site (microheterogeneity) thereby contributing to product homogeneity and consistency. Besides a recent patent application (WO2014195011A1) little/no attempts have been made that address differences in N‐glycosylation site occupancy on recombinant proteins (macroheterogeneity). Here, we present a strategy to overcome underglycosylation at N‐glycosylation sites on different recombinant glycoproteins when transiently expressed in *N. benthamiana*. We found that the expression of the single‐subunit oligosaccharyltransferase STT3D from *L. major* (LmSTT3D) substantially improves the N‐glycosylation efficiency on different transiently expressed recombinant glycoproteins.

## Results

### Recombinant IgG and an Fc‐fusion protein display considerable underglycosylation

**Figure 1:**
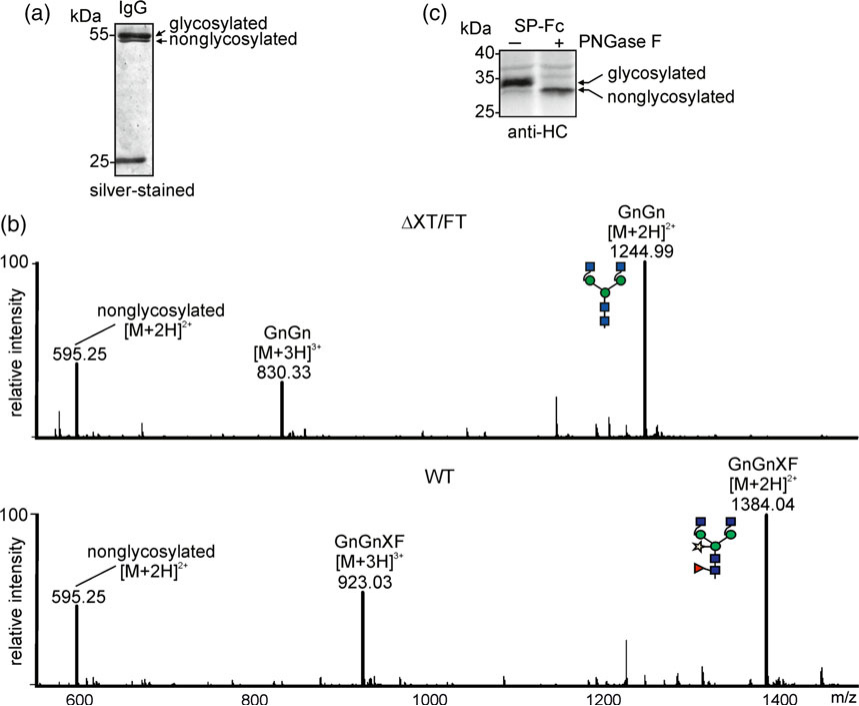
Underglycosylation is observed on transiently expressed IgG and on SP‐Fc. (a) A monoclonal IgG antibody was transiently expressed in *N. benthamiana* wild‐type plants. The IgG protein was purified 2 days after infiltration, separated by SDS‐PAGE and silver‐stained. The presence of glycosylated and nonglycosylated variants is indicated. The 25 kDa band represents the light chain. (b) A monoclonal antibody purified from *N. benthamiana* wild‐type (WT) or ΔXT/FT was digested with trypsin and subjected to LC‐ESI‐MS analysis. The mass [M + 2H]^2+^ of the nonglycosylated peptide EEQYNSTYR carrying the Fc‐N‐glycosylation site (Asn297) and the major glycosylated peaks [M + 2H]^2+^ and [M + 3H]^3+^ are depicted. Peak labels were made according to the ProGlycAn system (www.proglycan.com), and the glycan illustrations are drawn according to the nomenclature from the Consortium for Functional Glycomics. (c) SP‐Fc was expressed in *N. benthamiana* ΔXT/FT, and protein was extracted 24 h after infiltration and subjected to PNGase F digestion. Immunoblot detection was performed with anti‐IgG antibodies.

Previous studies have shown that the single N‐glycosylation site at position Asn297 from the heavy chain of different recombinant IgG molecules is frequently underglycosylated when transiently expressed in *N. benthamiana* (Bendandi *et al*., 2010; Loos *et al*., 2015; Strasser *et al*., 2008; Zeitlin *et al*., 2016) (Figure 1a). We expressed a monoclonal IgG antibody transiently in wild‐type as well as in the glyco‐engineered ΔXT/FT *N. benthamiana* line which is widely used as expression host for recombinant glycoproteins (Strasser *et al*., 2008, 2014). LC‐ESI‐MS analysis of the proteolytically digested heavy chain showed the presence of considerable amounts of the unglycosylated peptide in both expression hosts (Figure 1b). To investigate this variation in N‐glycosylation site occupancy more in detail and to better visualize the difference between glycosylated and nonglycosylated variants, we generated an expression construct where the Fc‐domain from the IgG heavy chain lacking a variable region is fused to a signal peptide for targeting to the secretory pathway (SP‐Fc). Upon SDS‐PAGE under reducing conditions and subsequent immunoblotting, the expressed SP‐Fc protein migrates at approximately 35 kDa and a faster migrating band at approximately 33 kDa is clearly detectable (Figure 1c). When digested with PNGase F which cleaves off the single N‐glycan, the deglycosylated band co‐migrates with the faster migrating band of the undigested SP‐Fc. Mass spectrometry‐based analysis of the glycosylation occupancy corroborates these findings for incomplete glycosylation (data not shown).

### LmSTT3D from the protist *Leishmania major* is retained in the ER of plants

**Figure 2:**
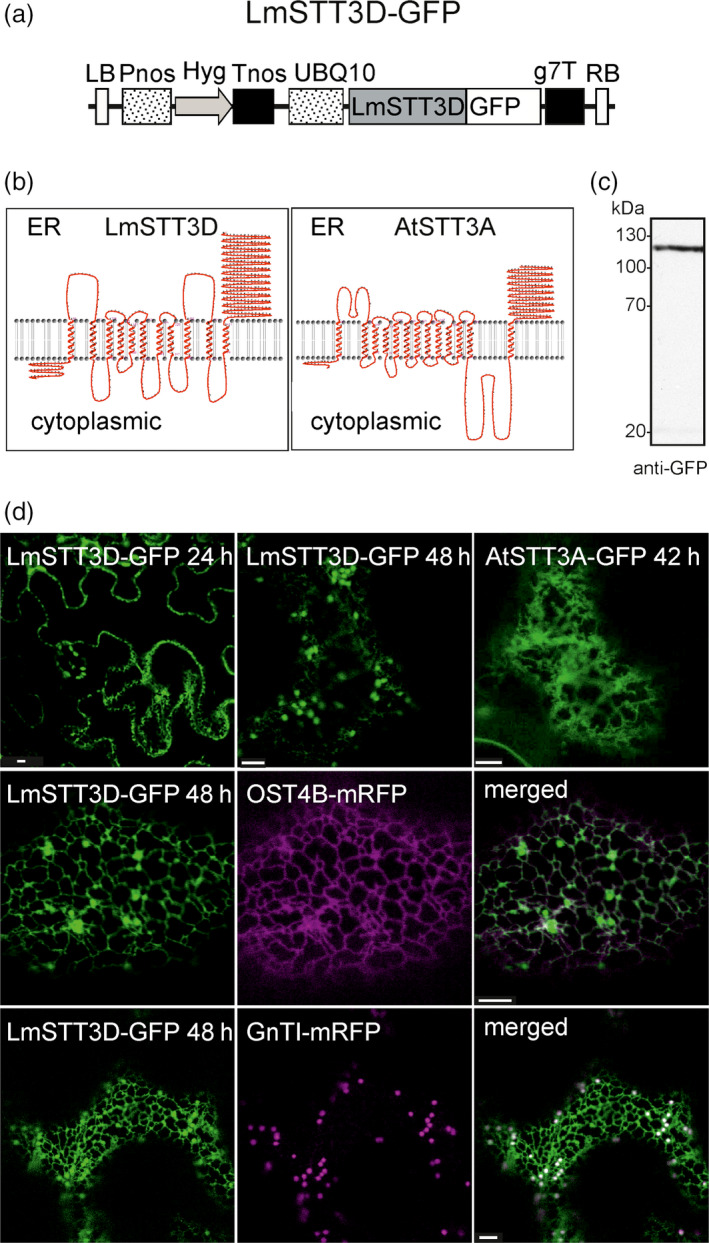
LmSTT3D‐GFP accumulates in the ER and Golgi. (a) Schematic representation of the UBQ10:LmSTT3D‐GFP expression vector. LB: left border; Pnos: nopaline synthase gene promoter; Hyg: hygromycin B phosphotransferase gene; Tnos: nopaline synthase gene terminator; UBQ10: *A. thaliana* ubiquitin‐10 promoter; LmSTT3D: *L. major* catalytic OST subunit STT3D open reading frame; GFP: green fluorescent protein; g7T: agrobacterium gene 7 terminator; RB: right border. (b) Topology of LmSTT3D and *A. thaliana* STT3A (AtSTT3A). The transmembrane domain regions and their topology were obtained using the HMMTop prediction program (http://www.enzim.hu/hmmtop/). The illustration was generated using TMRPres2D visualization (http://bioinformatics.biol.uoa.gr/TMRPres2D/). (c) Immunoblot (with anti‐GFP antibodies) of LmSTT3D‐GFP transiently expressed in *N. benthamiana*. (d) LmSTT3D‐GFP was either expressed alone or in combination with the ER‐marker OST4B‐mRFP and the Golgi‐marker GnTI‐mRFP in *N. benthamiana* leaf epidermal cells. Analysis of fluorescent proteins was carried out by confocal laser scanning microscopy at the indicated time after infiltration. Bars = 5 μm. Expression of AtSTT3A‐GFP is shown for comparison.

*Leishmania major* harbours four paralogues (termed LmSTT3A‐D) of the single‐subunit OST. In previous studies, it has been shown that the LmSTT3D from the protist *L. major* can rescue the growth and N‐glycosylation defects observed in *Saccharomyces cerevisiae* lacking a functional STT3 protein (Nasab *et al*., 2008) and improves N‐glycosylation efficiency of recombinant proteins expressed in *Pichia pastoris* (Choi *et al*., 2012). Consequently, we hypothesized that LmSTT3D activity may overcome the observed inefficient N‐glycosylation of IgG in our plant‐based expression system. To test this assumption, we generated a binary vector for expression of a codon‐optimized LmSTT3D variant fused to GFP (LmSTT3D‐GFP, Figure 2a) and transiently expressed the protein in *N. benthamiana*. According to the proposed topology model for LmSTT3D, the catalytic region close to the C‐terminus faces the lumen of the ER, similar to the predictions for *A. thaliana* STT3A (Figure 2b). On immunoblots, a single band of expected size is detectable for LmSTT3D‐GFP (Figure 2c). In wild‐type leaf epidermal cells, ER‐labelling was visible under the confocal microscope 1 day after infiltration (Figure 2d). Two and 3 days after infiltration, ER and additional puncta were detectable which represent Golgi bodies as well as undefined vesicular structures. In contrast to that, *A. thaliana* STT3A‐GFP was only observed in the ER. Co‐localization with the ER‐resident OST4B‐mRFP, a subunit of the plant oligosaccharyltransferase complex (Farid *et al*., 2013), or the *cis*/medial Golgi‐marker GnTI‐mRFP (Schoberer *et al*., 2013) confirmed the subcellular localization of LmSTT3D‐GFP (Figure 2d) suggesting that LmSTT3D‐GFP is incompletely retained in the ER.

### LmSTT3D enhances the N‐glycosylation occupancy of recombinant SP‐Fc and IgG

**Figure 3:**
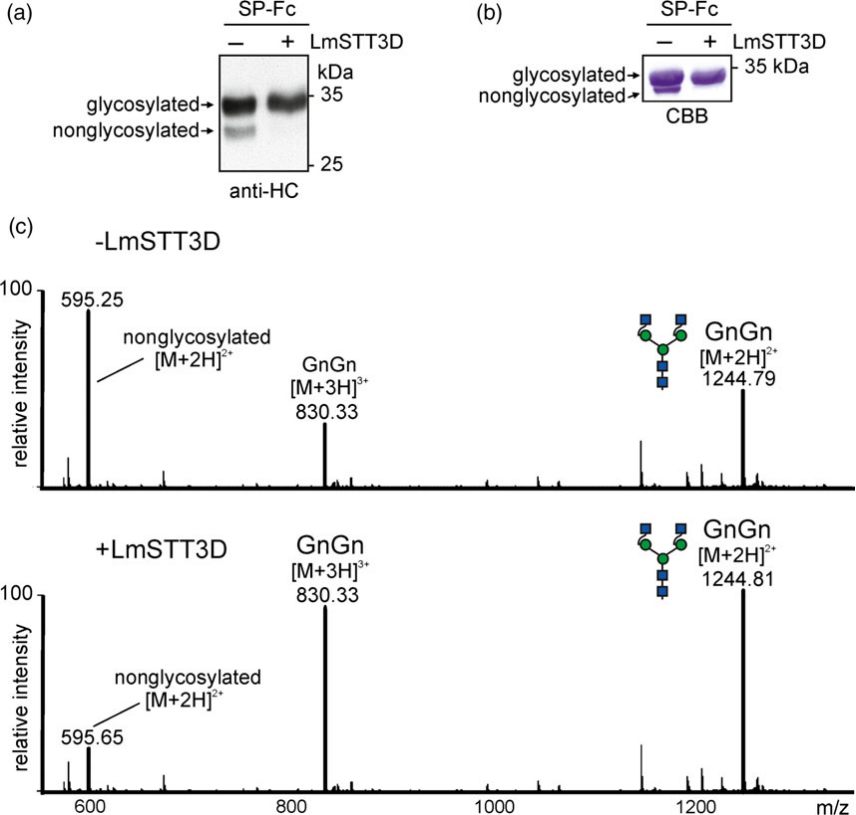
LmSTT3D‐GFP co‐expression increases the N‐glycosylation site occupancy on SP‐Fc. SP‐Fc was transiently expressed in *N. benthamiana* leaves together with UBQ10:LmSTT3D‐GFP. (a) Proteins were extracted from wild‐type 2 days postinfiltration and subjected to SDS‐PAGE and immunoblotting using anti‐IgG heavy chain (HC) antibodies. (b) SDS‐PAGE and Coomassie Brilliant Blue staining of SP‐Fc purified from ΔXT/FT. (c) LC‐ESI‐MS analysis of tryptic glycopeptides from SP‐Fc expressed in ΔXT/FT. In the shown spectra, the peak at 595.25 ([M + 2H]^2+^) is assigned to the nonglycosylated peptide EEQYNSTYR, and the peaks at 830.33 ([M + 3H]^3+^) and 1244.8 ([M + 2H]^2+^) are assigned to the complex N‐glycan GnGn (nomenclature according to the ProGlycAn system: www.proglycan.com).

**Figure 4:**
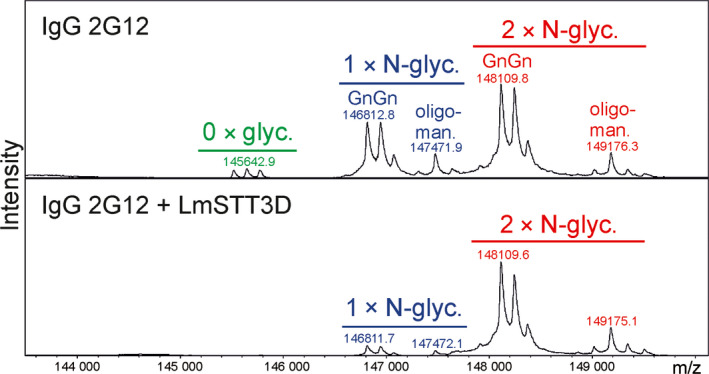
The N‐glycan site occupancy of fully assembled IgG in the presence or absence of LmSTT3D‐GFP was determined using LC‐ESI‐MS. The peaks corresponding to unglycosylated (green), hemi‐glycosylated (blue, one N‐glycan) and fully glycosylated (red, two N‐glycans) IgG (HIV‐neutralizing antibody 2G12) are highlighted. Multiple peaks represent different glycoforms (complex N‐glycan GnGn, oligomannosidic glycans) and variations in the clipping of C‐terminal lysine. Please note, the clipping of lysine is not found on variants carrying oligomannosidic N‐glycans indicating that this processing reaction occurs in a post‐ER compartment.

In the next experiments, we examined whether LmSTT3D‐GFP can improve the N‐glycosylation efficiency of SP‐Fc and IgG when transiently co‐expressed in *N. benthamiana*. In the presence of LmSTT3D‐GFP, the faster migrating band of SP‐Fc disappeared, indicating an enhanced occupancy of glycosylation site Asn297 (Figure 3a and b). MS‐based analysis of peptides/glycopeptides derived from proteolytically digested SP‐Fc expressed in ΔXT/FT demonstrated that the co‐expression of LmSTT3D‐GFP drastically reduced the amount of the nonglycosylated variant (Figure 3c, Table 1). The N‐glycan composition of the recombinantly expressed proteins was not altered upon LmSTT3D‐GFP co‐expression. The major N‐glycan peak corresponds to processed complex N‐glycans (GnGn: GlcNAc_2_Man_3_GlcNAc_2_) (Figure 3c) indicating that LmSTT3D‐GFP co‐expression does not interfere with complex N‐glycan processing of SP‐Fc in the Golgi. The same result was obtained for an IgG co‐expressed with LmSTT3D‐GFP (Figure S1 and Table 1). Intact MS analysis of the fully assembled IgG 2G12 revealed further that in the absence of LmSTT3D‐GFP, nonglycosylated as well as hemi‐glycosylated (only one of the two heavy chains carries an N‐glycan) forms are present. Co‐expression of LmSTT3D‐GFP leads to an increase in fully assembled IgG with two N‐glycans, one attached to each heavy chain (Figure 4). In summary, our data show that LmSTT3D‐GFP co‐expression increases the N‐glycosylation site occupancy of SP‐Fc and IgG.

**Table 1:** Comparison of the LmSTT3D‐GFP effect on N‐glycosylation of recombinant glycoproteins expressed in ΔXT/FT

Protein	N‐glycosylation site (GS)	% glycosylated	% glycosylated + LmSTT3D	% increase	Number of repetitions
Fc	GS1 NST	56 ± 3	93 ± 2	66	3
IgG	GS1 NST	87 ± 5	98 ± 4	13	3
IgE	GS3 NKT	20 ± 7	36 ± 3	80	2
	GS5 NLT	40 ± 11	91 ± 3	128	
	GS6 NHS	<2 ± 3	63 ± 4	>1000	
IgA1	GS1 NLT	96 ± 0	94 ± 4		2
	GS2 NVS	59 ± 1	95 ± 4	60	
EPO‐Fc	GS1 NIT	60 ± 5	81 ± 3	35	2
	GS3 NSS	95 ± 2	93 ± 0		
	GS4 NST	90 ± 1	99 ± 2	10	
IFN‐γ	GS1 + GS2	20 ± 7	67 ± 12	235	5

Mean values + standard deviation from independent experiments (biological replicates) are shown. The glycosylation site occupancy of IFN‐γ was calculated by quantification of bands from immunoblots. All other values are derived from MS‐based quantification of peptides from purified proteins. Please note, due to an incomplete proteolytic digestion, no reliable quantification of GS2 from EPO‐Fc could be performed.

### LmSTT3D improves the N‐glycosylation efficiency of different recombinant glycoproteins

**Figure 5:**
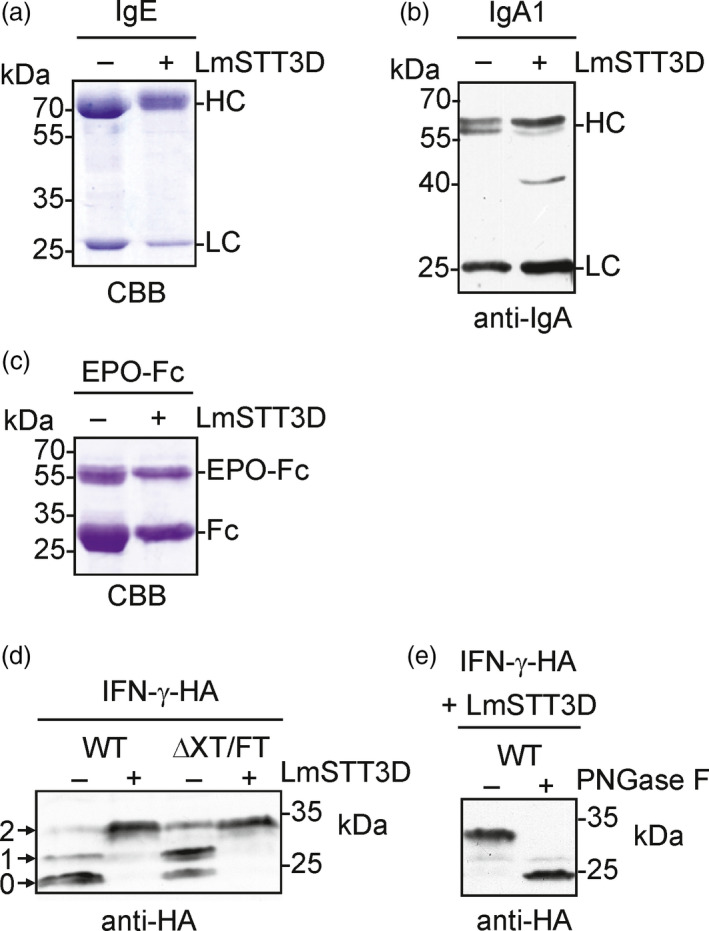
LmSTT3D‐GFP enhances the N‐glycosylation efficiency of different recombinant proteins when transiently expressed in *N. benthamiana*. (a) Human IgE was expressed in ΔXT/FT in the presence (+) or absence (−) of LmSTT3D‐GFP, and the purified IgE was analysed by SDS‐PAGE and Coomassie Brilliant Blue (CBB) staining. (b) Human IgA1 was expressed in ΔXT/FT, and total protein extracts were analysed by immunoblotting with antibodies against the alpha heavy chain and the kappa light chain (anti‐IgA). (c) EPO‐Fc was expressed in ΔXT/FT, purified and subjected to SDS‐PAGE and CBB staining. (d) Expression of IFN‐γ‐HA in the presence (+) or absence (−) of LmSTT3D‐GFP. Protein extracts were subjected to SDS‐PAGE and immunoblotting using anti‐HA antibodies. The migration position of the nonglycosylated (0), mono‐ (1) and di‐glycosylated (2) IFN‐γ‐HA protein is indicated. (e) PNGase F digestion of IFN‐γ‐HA co‐expressed with LmSTT3D‐GFP.

We found that LmSTT3D‐GFP co‐expression is a suitable tool to increase the N‐glycosylation efficiency of SP‐Fc and IgG. To extend our findings, we tested the impact of LmSTT3D‐GFP on the N‐glycosylation site occupancy of other transiently expressed mammalian glycoproteins carrying multiple glycosylation sites (GS). First, we co‐expressed recombinant IgE (7 GS) and IgA1 (2 GS) together with LmSTT3D‐GFP. These immunoglobulins have been recently expressed in *N. benthamiana* and contain N‐glycosylation sites that were partially occupied (Göritzer *et al*., 2017; Montero‐Morales *et al*., 2017). GS1, GS2, GS4 and GS7 are fully occupied on recombinant IgE. GS3 and GS5, on the other hand, display partial glycosylation and GS6 is normally not occupied (Montero‐Morales *et al*., 2017; Plomp *et al*., 2014). In the presence of LmSTT3D‐GFP, we observed a slight shift in the migration position of the IgE heavy chain (Figure 5a). MS‐based analysis of IgE glycosylation sites confirmed an increase in N‐glycosylation site occupancy for the IgE glycosylation sites that were previously found to be incompletely glycosylated in the absence of LmSTT3D‐GFP (80% increase for GS3 and 128% for GS5, Table 1). Interestingly, GS6 becomes N‐glycosylated in the presence of LmSTT3D‐GFP and more than half of the purified IgE is now glycosylated at this particular site (Table 1). The N‐glycans found on IgE GS6 were mainly processed complex type N‐glycans that are commonly found on plant‐produced recombinant glycoproteins indicating that the LmSTT3D‐mediated transfer does not lead to altered N‐glycan processing (Figure S2). GS2 at the C‐terminus of IgA1 is normally incompletely N‐glycosylated when expressed in plants or human cells (Göritzer *et al*., 2017). In the presence of LmSTT3D‐GFP, however, we detected a reduction in the incompletely glycosylated alpha heavy chain from IgA1 (Figure 5b) and MS‐based quantification revealed almost complete glycosylation of the sequon in the C‐terminal tailpiece (Table 1) without affecting N‐glycan processing (Figure S3).

To see whether the positive effect of LmSTT3D can also be observed with recombinant glycoproteins that are not related to immunoglobulins, we transiently expressed human EPO‐Fc (Castilho *et al*., 2011) and the cytokine interferon‐γ (IFN‐γ). Differences in SDS‐PAGE migration of EPO‐Fc were observed when LmSTT3D‐GFP was co‐expressed indicating a reduction in underglycosylation (Figure 5c). Quantification of glycopeptides derived from EPO showed a clear increase at glycosylation site one (Table 1). As observed for Fc glycosylation, the N‐glycan profiles were virtually identical in the absence or presence of LmSTT3D‐GFP. Mainly the fully processed GnGn glycans were present on all three N‐glycosylation sites of EPO as well as on the Fc site when expressed in ΔXT/FT (Figure S4 and data not shown), suggesting that LmSTT3D‐GFP expression does not interfere with N‐glycan processing.

The effect of LmSTT3D on the N‐glycosylation site occupancy of IFN‐γ which carries two N‐glycosylation sites (Asn25 and Asn97, Figure S5) was examined by immunoblots of a variant carrying a C‐terminal HA‐tag. IFN‐γ‐HA shows three bands on immunoblots indicating that it is incompletely glycosylated (no N‐glycan, a single N‐glycan or fully glycosylated with two N‐glycans) when transiently expressed in *N. benthamiana* wild‐type and ΔXT/FT. Co‐expression of LmSTT3D‐GFP resulted in the appearance of a major protein band representing the fully glycosylated protein that could be converted to the nonglycosylated IFN‐γ‐HA by PNGase F digestion (Figure 5d and e, Table 1). These data show that LmSTT3D co‐expression improves the N‐glycosylation site occupancy of numerous glycoproteins.

### LmSTT3D‐GFP‐HDEL is efficiently retained in the ER

**Figure 6:**
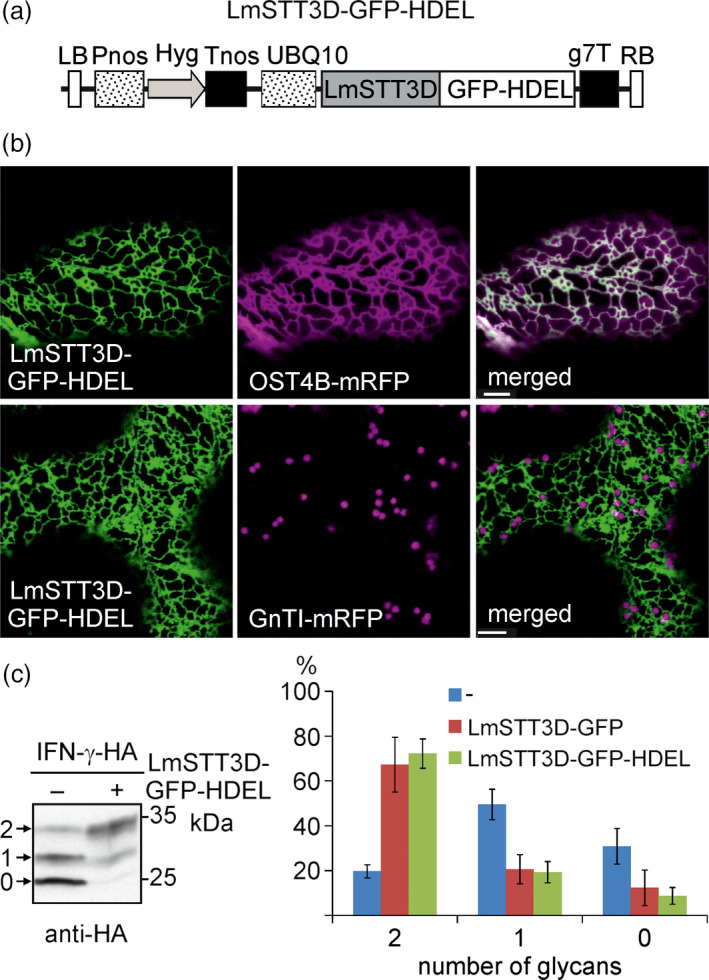
Attachment of the HDEL tetrapeptide improves ER localization of LmSTT3D‐GFP and does not interfere with its functionality. (a) Schematic illustration of the UBQ10:LmSTT3D‐GFP‐HDEL expression vector. For abbreviations, see legend of Figure 2. (b) LmSTT3D‐GFP‐HDEL was either expressed alone or in combination with the ER‐marker OST4B‐mRFP in wild‐type leaf epidermal cells. Images were acquired two days postinfiltration. Bars = 5 μm. (c) SDS‐PAGE and immunoblotting of IFN‐γ‐HA expressed in ΔXT/FT in the presence (+) or absence (−) of LmSTT3D‐GFP‐HDEL. Quantification of IFN‐γ‐HA protein bands upon expression in ΔXT/FT. The diagram shows mean values plus standard deviation from at least five biological replicates.

While the co‐expression of LmSTT3D‐GFP resulted in a significant improvement of the N‐glycosylation efficiency on various proteins, the overlapping occurrence in the ER and Golgi bodies suggests that part of the protein is not functional due to the mislocalization. Consequently, we examined whether a LmSTT3D variant with increased ER accumulation improves its functionality. Thus, a construct was generated which expressed LmSTT3D‐GFP with a C‐terminal HDEL tetrapeptide for ER retrieval (Figure 6a). Imaging by confocal microscopy showed that the attachment of the HDEL motif leads to an efficient steady‐state distribution of LmSTT3D‐GFP‐HDEL in the ER in *N. benthamiana* leaf epidermal cells (Figure 6b). No signal was observed in Golgi bodies. To analyse the functionality of LmSTT3D‐GFP‐HDEL, we co‐expressed it together with IFN‐γ‐HA and analysed the glycosylation status. Like LmSTT3D‐GFP, the LmSTT3D‐GFP‐HDEL variant was functional and improved the N‐glycosylation site occupancy of IFN‐γ‐HA. Quantification of bands from immunoblots showed no difference between LmSTT3D‐GFP and LmSTT3D‐GFP‐HDEL (Figure 6c) suggesting that the expression of the fully ER‐retained LmSTT3D‐GFP‐HDEL variant does not increase N‐glycosylation efficiency compared to the incompletely retained variant.

## Discussion

N‐glycans have a strong impact on protein folding, stability and influence the interaction with other proteins. N‐glycosylation of recombinant biopharmaceuticals is critical for product quality (Reusch and Tejada, 2015). For example, the single N‐glycan from the IgG Fc‐domain modulates immune effector functions and unglycosylated IgG variants display drastically reduced affinity for Fcγ‐receptors (Ferrara *et al*., 2011; Jefferis and Lund, 2002; Shields *et al*., 2002). Moreover, the presence of additional N‐glycans can improve the *in vivo* half‐life and activity of recombinant biopharmaceuticals. This has been impressively demonstrated for a hyperglycosylated EPO variant (darbepoetin alfa) that is glycosylated at two additionally introduced N‐glycosylation sites and has been approved for treatment of anaemia (Elliott *et al*., 2003). EPO or IgG from human serum and recombinant variants thereof expressed in mammalian cells are typically very efficiently glycosylated (Table S1). By contrast, *N. benthamiana* (Table 1) and to a certain degree also other expression systems such as *P. pastoris* (Choi *et al*., 2012) or insect cells (Sareneva *et al*., 1995) display more variation in N‐glycosylation efficiency. Despite the documented importance of proper N‐glycosylation site occupancy, comparably few studies have so far approached the diversity caused by the absence of glycans at particular sites on recombinant glycoproteins. Previous studies have shown that LmSTT3D, the single catalytic subunit from the protozoan *L. major*, can replace the function of the endogenous STT3 subunit from *S. cerevisiae* and complements growth and N‐glycosylation defects associated with OST deficiency (Nasab *et al*., 2008). Biochemical and genetic evidence indicates that LmSTT3D is functionally independent and not integrated into the native OST complex when heterologously expressed (Hese *et al*., 2009; Nasab *et al*., 2008).

Recombinant IFN‐γ has been approved for treatment of different human diseases and is a promising candidate for cancer immunotherapy (Razaghi *et al*., 2016). Glycosylation of IFN‐γ is important for its proteolytic stability, secretion and circulatory half‐life (Bocci *et al*., 1985; Sareneva *et al*., 1993, 1995, 1996). Transient expression in *N. benthamiana* indicates that IFN‐γ is inefficiently glycosylated in the absence of LmSTT3D. We currently do not know whether both sites are equally affected or whether Asn97, which is present in an α‐helical region, is less occupied as has been suggested for IFN‐γ from human cells (Sareneva *et al*., 1996). In contrast to *N. benthamiana*, approximately two‐thirds of native human IFN‐γ is fully glycosylated (Rinderknecht *et al*., 1984; Sareneva *et al*., 1995) and recombinant IFN‐γ expressed in CHO fed‐batch cultures displays low amounts of nonglycosylated protein (Wong *et al*., 2010). The reason for this discrepancy in N‐glycosylation efficiency between mammalian cells and plants is currently unknown, but may reflect differences in the composition and function of the OST complex. Notably, in the presence of LmSTT3D, similar levels of fully glycosylated IFN‐γ are obtained in plants and on the naturally occurring protein (Table 1 and Table S1).

We found that not all analysed N‐glycosylation sites were equally well glycosylated upon LmSTT3D co‐expression. Glycosylation of GS3 from EPO‐Fc or GS1 from IgA1, which were already efficiently occupied in the absence of LmSTT3D, was not improved. On the other hand, GS2 from IgA1, which is only partially glycosylated when expressed in plants or human cells (Göritzer *et al*., 2017), could be completely modified with N‐glycans upon LmSTT3D expression. This site is likely post‐translationally modified in mammalian cells and plays an important role in the assembly of dimeric IgA1 (Atkin *et al*., 1996). Consequently, our data indicate that LmSTT3D preferentially glycosylates certain N‐glycosylation sites which has also been recognized in a previous study (Nasab *et al*., 2008). The precise sequence or conformational constraints influencing LmSTT3D‐dependent glycosylation are unknown. Remarkably, the co‐expression of LmSTT3D resulted in the glycosylation of IgE GS6. This is in contrast to native serum or recombinantly produced IgE from human cells (Montero‐Morales *et al*., 2017; Plomp *et al*., 2014), indicating that LmSTT3D has a more relaxed substrate specificity and recognizes glycosylation sites that are normally not used by the mammalian OST complex. Due to the various biological roles of N‐glycans, the functional relevance of an additional N‐glycan is difficult to predict. LmSTT3D co‐expression facilitates the production of non‐natural glycoproteins that can be used to test the influence on physicochemical properties of proteins and known protein interactions in future studies.

A further increase in the glycosylation efficiency may be achieved by stable expression of LmSTT3D in *N. benthamiana* or incorporation of LmSTT3D into multicassette expression vectors used for transient expression together with a glycoprotein of interest to ensure that all cells express LmSTT3D. The stable expression of the KDEL tagged variant, which is at least equally functional when expressed with IFN‐γ or IgG (data not shown), will less likely interfere with N‐glycan processing in the Golgi or overall Golgi organization and function.

Apart from the expression levels, interaction with ER‐resident proteins, polypeptide substrate specificity or enzyme kinetics of the catalytic STT3 subunit, glycosylation efficiency may be controlled by supply of the preassembled lipid‐linked oligosaccharide substrate. Deprivation of glucose from CHO cell cultures reduced the amounts of lipid‐linked oligosaccharides resulting in the expression of nonglycosylated monoclonal antibodies (Liu *et al*., 2014). In addition to optimized metabolic parameters, a limitation in donor substrate availability may be the result of inefficient lipid‐linked oligosaccharide transfer into the ER. This shortcoming may be overcome by co‐expression of an artificial flippase (Parsaie Nasab *et al*., 2013). Moreover, it is well known that protein intrinsic structural constraints strongly influence the N‐glycosylation efficiency at distinct sites. For example, the presence of a serine instead of a threonine in the consensus site N‐X‐S/T is less preferred by the OST complex and N‐X‐T sites are more frequently glycosylated in organisms from different eukaryotic domains of life (Zielinska *et al*., 2012). Exchange of amino acids in the sequon or at adjacent sites of the polypeptide can drastically alter the glycosylation site occupancy (Murray *et al*., 2015). For a recombinant elastase expressed in *P. pastoris,* a change of the sequon from N‐X‐S to N‐X‐T resulted in an increased glycosylation efficiency that was accompanied by higher production levels of the recombinant glycoprotein (Han *et al*., 2015). By contrast, mutagenesis of flanking amino acids and generation of an optimized aromatic sequon with increased glycosylation efficiency negatively affected the secretion of IFN‐γ expressed in human cells and caused variability in protein expression of another glycoprotein (Huang *et al*., 2017). Likewise, antibody engineering by generation of an aromatic sequon (FANST instead of the canonical QYNST) improved the thermal stability of the antibody, but reduced the affinity to specific Fcγ‐receptors (Chen *et al*., 2016). These studies highlight impressively that protein engineering at glycosylation sites can have various consequences leading to reduced productivity or altered product quality. Consequently, strategies aiming at an improvement of N‐glycosylation by engineering of the OST complex are very promising and relevant for different plant‐based expression platforms (Hamorsky *et al*., 2015; Rademacher *et al*., 2008; Vamvaka *et al*., 2016; Van Droogenbroeck *et al*., 2007). Further advances require a better understanding of the OST complex composition and molecular function of the individual subunits. Taken together, our findings demonstrate that transient LmSTT3D expression is a robust extension of currently existing glyco‐engineering approaches and should be integrated into production processes to reduce product heterogeneity and improve biological activities related to N‐glycosylation of recombinant glycoproteins.

## Experimental procedures

### Cloning of expression vectors

The expression constructs for IgG 2G12 (Schähs *et al*., 2007), EPO‐Fc (Castilho *et al*., 2011), IgE (Montero‐Morales *et al*., 2017) and IgA1 (Göritzer *et al*., 2017) were described previously. To generate the SP‐Fc expression vector, the DNA fragment coding for GCSI‐CTS‐Fc was amplified from GCSI‐CTS‐GFPglyc (Schoberer *et al*., 2009) by PCR using primers GCSI‐7F (TATATCTAGAATGACCGGAGCTAGCCGTCGGAGC) and Fc‐6R (TATACTCGAGTTATTTACCCGGAGACAGGGAGAGG). The PCR product was digested with XbaI/XhoI and cloned into XbaI/SalI sites of p47 (Hüttner *et al*., 2014) to generated p71‐GCSI‐Fc. Subsequently, the chitinase signal peptide was amplified from *N. benthamiana* cDNA by PCR using Nb‐Chi‐F1 (TATATCTAGAATGAGGCTTAGAGAATTCACAG) and Nb‐Chi‐R2 (TATAGGATCCTGCCGAGGCAGAGAGTAGGAGAGA), XbaI/BamHI digested and cloned into XbaI/BamHI digested p71‐GCSI‐Fc, resulting in p71‐SP‐Fc. For IFN‐γ expression, a codon‐optimized DNA fragment encoding human IFN‐γ was synthetized by GeneArt Gene Synthesis (Thermo Fisher Scientific). The synthetic DNA fragment was XbaI/BamHI digested and cloned into the XbaI/BamHI sites of expression vector p43. The vector p43 is a derivative of expression vector p27 (Strasser *et al*., 2007) whereby the CaMV 35S promoter was replaced by the *A. thaliana* UBQ10 promoter and a sequence encoding a 3x HA‐tag for C‐terminal fusion was inserted upstream of the terminator sequence (Figure S5). To generate the LmSTT3D‐GFP expression vector a codon‐optimized open reading frame coding for *L. major* STT3D (Nasab *et al*., 2008) was synthetized by GeneArt Gene Synthesis. The LmSTT3D open reading frame was excised by XbaI/BamHI digestion and cloned into XbaI/BamHI sites of p47 or p56. Vector p56 is derived from p47 by replacement of the GFP coding sequence with the one for GFP‐HDEL. For generation of the STT3A‐GFP expression vector p20‐STT3A, the *A. thaliana* STT3A coding region was amplified by PCR as described previously (Farid *et al*., 2013) and cloned into XbaI/BamHI digested plasmid p20F (Schoberer *et al*., 2009).

### Transient expression and immunoblot analysis

All plant expression vectors were transformed into *Agrobacterium tumefaciens* (strain UIA143) (Farrand *et al*., 1989). Syringe‐mediated agroinfiltration was used for transient expression in leaves of 4‐ to 5‐week‐old *N. benthamiana* grown on soil under long‐day conditions (16 h light/8 h dark) at 25°C. At the indicated time points, leaf pieces were harvested from infiltrated plants, and total protein extracts were prepared and subjected to SDS‐PAGE followed by silver staining (Strasser *et al*., 2004) or immunoblotting as described in detail previously (Shin *et al*., 2017). IgG and Fc‐containing fragments were monitored with anti‐human IgG (H+L)‐horseradish peroxidase antibody (Promega, Mannheim, Germany), IgA with anti‐alpha chain/anti‐kappa‐chain antibodies and IFN‐γ‐HA with anti‐HA antibodies. For deglycosylation, protein extracts were incubated with peptide‐N‐glycosidase F (PNGase F) (New England Biolabs, Frankfurt am Main, Germany) according to the manufacturer's procedure. Quantification of gel bands on immunoblots was performed with a ChemiDoc imager (Bio‐Rad, Vienna, Austria) and Quantity One 1D analysis software (Bio‐Rad).

For detection of LmSTT3D‐GFP on immunoblots, leaf material was harvested 48 h after infiltration of *N. benthamiana* leaves. Proteins were extracted with 1 × Laemmli sample buffer supplemented with 6M urea and incubated at 37°C for 5 min. The fusion protein was detected with anti‐GFP horseradish peroxidase (MACS Miltenyi Biotec, Bergisch Gladbach, Germany) antibodies.

### Confocal imaging of fluorescent protein fusions

Leaves of 4‐ to 5‐week‐old *N. benthamiana* were infiltrated with agrobacterium suspensions carrying binary plant expression vectors for expression of GFP‐ or mRFP‐tagged proteins with the following optical densities (OD_600_): 0.1 for p47‐LmSTT3D (LmSTT3D‐GFP), p56‐LmSTT3D (LmSTT3D‐GFP‐HDEL), p20‐STT3A (AtSTT3A‐GFP). Agrobacteria carrying the expression constructs p31‐OST4B (OST4B‐mRFP, ER‐marker) (Farid *et al*., 2013) and p31‐GnTI (GnTI‐mRFP, Golgi‐marker) (Schoberer *et al*., 2013) were infiltrated with OD_600_ = 0.05. Confocal images were acquired 1 and 2 days postinfiltration on a Leica SP5 II confocal microscope using the Leica LAS AF software system (http://www.leica.com). Dual‐colour image acquisition of cells expressing both GFP and mRFP was performed simultaneously. Postacquisition image processing was performed in Adobe PHOTOSHOP CS5.

### LC‐ESI‐MS analysis

The full‐length heavy chain from IgGs and SP‐Fc was purified from the protein extract by binding to rProtein A Sepharose™ Fast Flow (GE Healthcare Europe, Vienna, Austria). Purified protein was subjected to SDS‐PAGE under reducing conditions and Coomassie Brilliant Blue staining. The corresponding protein band was excised from the gel, destained, carbamidomethylated, in‐gel trypsin digested and analysed by liquid chromatography electrospray ionization mass spectrometry (LC‐ESI‐MS), as described in detail previously (Stadlmann *et al*., 2008). A detailed explanation of N‐glycan abbreviations can be found at http://www.proglycan.com. Protein purification and MS‐based analysis of (glyco)peptides from EPO‐Fc, IgE and IgA1 were described in detail recently (Castilho *et al*., 2011; Göritzer *et al*., 2017; Montero‐Morales *et al*., 2017). Site occupancy was calculated from the peak area of nonglycosylated versus the sum of the peak areas of all glycoforms including relevant adduct ions and observed charged states. The principal suitability of this ‘peak sum’ approach under the conditions applied was verified in two stages. First, selected samples were subjected to deglycosylation with peptide‐N‐glycosidase A (ProGlycAn, Vienna, Austria) and the ratio of the Asn vs. the Asp containing glycopeptides, which separate in RP‐HPLC, was measured. Peptides differing in charged amino acids may have differing mass spectrometric responses, and hence, in a second stage, the Fc tandem peptide EEQYNSTYREEQYDSTYR (JP peptides, Berlin, Germany) was digested with trypsin to obtain an equimolar mixture of the Asn and the Asp form of the Fc (glyco‐)peptide. These measurements showed that the ‘peak sum’ approach gave reliable values with a possible overestimation of nonglycosylation of a very few percentages especially in the case of low underglycosylation. We assume the situation for other glycoproteins to be comparable to that with IgG Fc.

### Mass spectrometric analysis of fully assembled IgGs

The purified IgGs were directly injected to a LC‐ESI‐MS system (LC: Dionex Ultimate 3000 LC). A gradient from 20% to 80% acetonitrile in 0.05% trifluoroacetic acid (using a Thermo ProSwift™ RP‐4H column (0.2 × 250 mm)) at a flow rate of 8 μL/min was applied (30‐minute gradient time). Detection was performed with a Q‐TOF instrument (Bruker maXis 4G) equipped with the standard ESI source in positive ion, MS mode (range: 750–5000 Da). Instrument calibration was performed using ESI calibration mixture (Agilent). Data were processed using Data Analysis 4.0 (Bruker), and the spectrum was deconvoluted by MaxEnt.

## Conflict of interest

The authors declared that they have no conflict of interests.

## Supporting information


**Figure S1** Mass spectra of the IgG glycopeptide in the presence or absence of LmSTT3D‐GFP.
**Figure S2** Mass spectra of IgE glycopeptides in the presence or absence of LmSTT3D‐GFP.
**Figure S3** Mass spectra of IgA1 glycopeptides in the presence or absence of LmSTT3D‐GFP.
**Figure S4** Mass spectra of the EPO‐Fc glycopeptides harbouring glycosylation site 1 in the presence or absence of LmSTT3D‐GFP.
**Figure S5** Schematic illustration of the IFN‐γ‐HA expression construct and the corresponding amino acid sequence.

**Table S1** Comparison of the N‐glycosylation site occupancy of native and recombinant glycoproteins.
