# Supplementary material for: An oligosaccharyltransferase from Leishmania major increases the N‐glycan occupancy on recombinant glycoproteins produced in Nicotiana benthamiana
Source: Plant Biotechnol J. 2018 Mar 25;16(10):1700–9. doi: 10.1111/pbi.12906 (PMC6131413; doi:10.1111/pbi.12906)
Supplement: Supplementary file 1 — Figure S1 Mass spectra of the IgG glycopeptide in the presence or absence of LmSTT3D‐GFP. Figure S2 Mass spectra of IgE glycopeptides in the presence or absence of LmSTT3D‐GFP. Figure S3 Mass spectra of IgA1 glycopeptides in the presence or absence of LmSTT3D‐GFP. Figure S4 Mass spectra of the EPO‐Fc glycopeptides harbouring glycosylation site 1 in the presence or absence of LmSTT3D‐GFP. Figure S5 Schematic illustration of the IFN‐γ‐HA expression construct and the corresponding amino acid sequence. [file PBI-16-1700-s001.pdf]

EEQYNSTYR (1189.5120 Da)

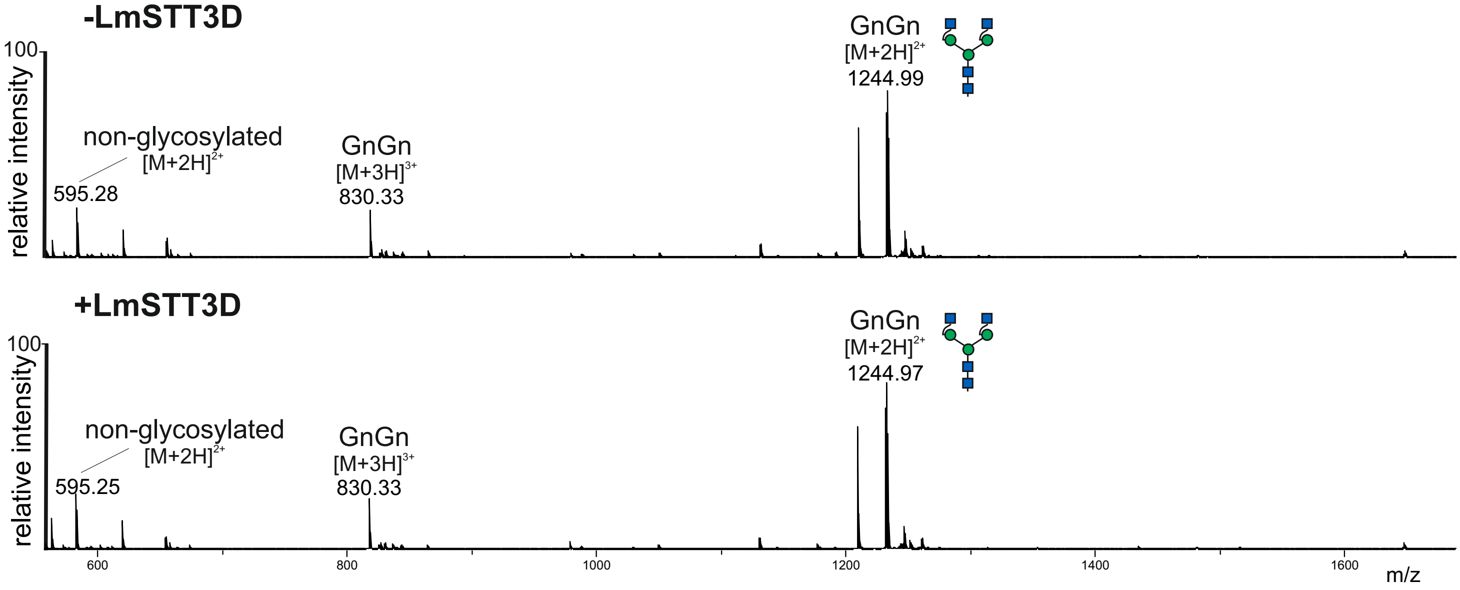

Figure S1 Mass spectra of (glyco)peptides from IgG. IgG was transiently expressed in *N. benthamiana* ΔXT/FT in the presence or absence of LmSTT3D-GFP. Purified IgG was trypsin digested and analysed by LC-ESI-MS.

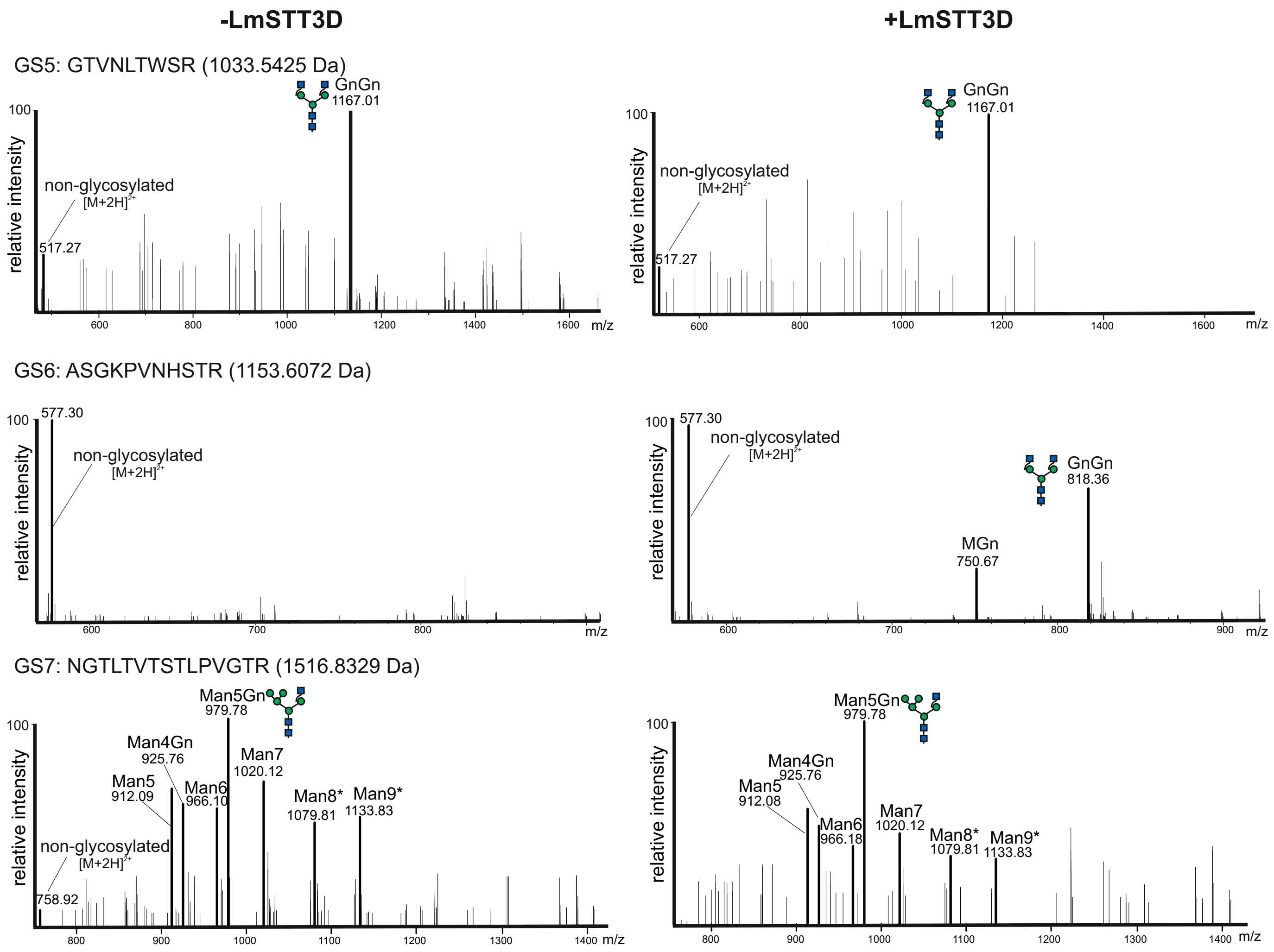

Figure S2 Mass-spectra of IgE (glyco)peptides harbouring glycosylation sites (GS) 5, 6 and 7. IgE was transiently expressed in *N. benthamiana* ΔXT/FT in the presence or absence of LmSTT3D-GFP. Purified IgE was trypsin digested and analysed by LC-ESI-MS. The mass range displays the major glycoforms. Except for the glycopeptides harbouring GS5, the peaks corresponding to triple charged glycopeptides ($[M+3H]^{3+}$) are indicated. The major glycoform on GS5 as well as all non-glycosylated peptides are shown in their double charged ($[M+2H]^{2+}$) state. Ammonia adducts are denoted by an asterisk.

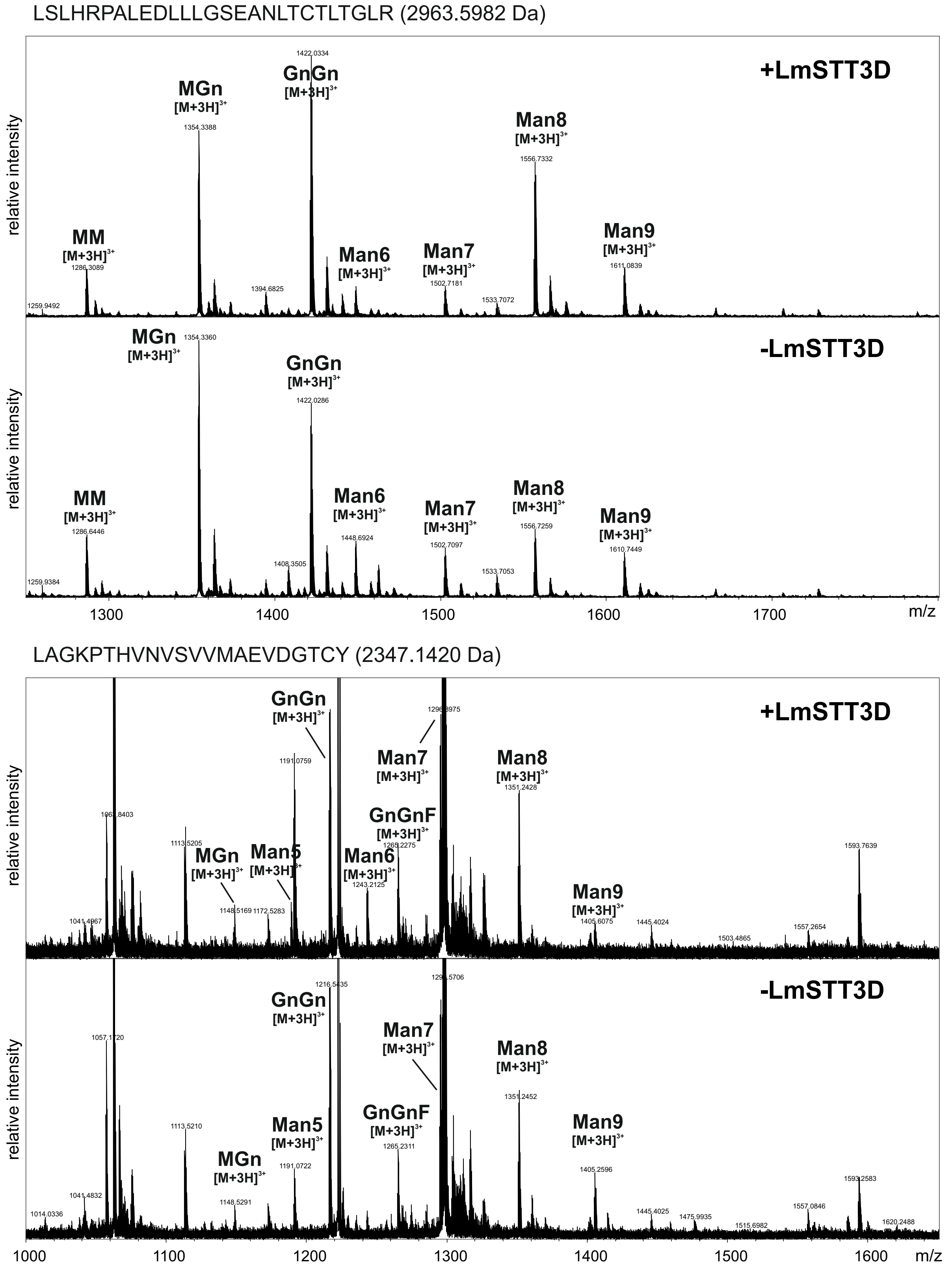

Figure S3 Mass-spectra of IgA1 (glyco)peptides. IgA1 was transiently expressed in *N. benthamiana* ΔXT/FT in the presence or absence of LmSTT3D-GFP. The purified IgA1 heavy chain was trypsin digested and analysed by LC-ESI-MS.

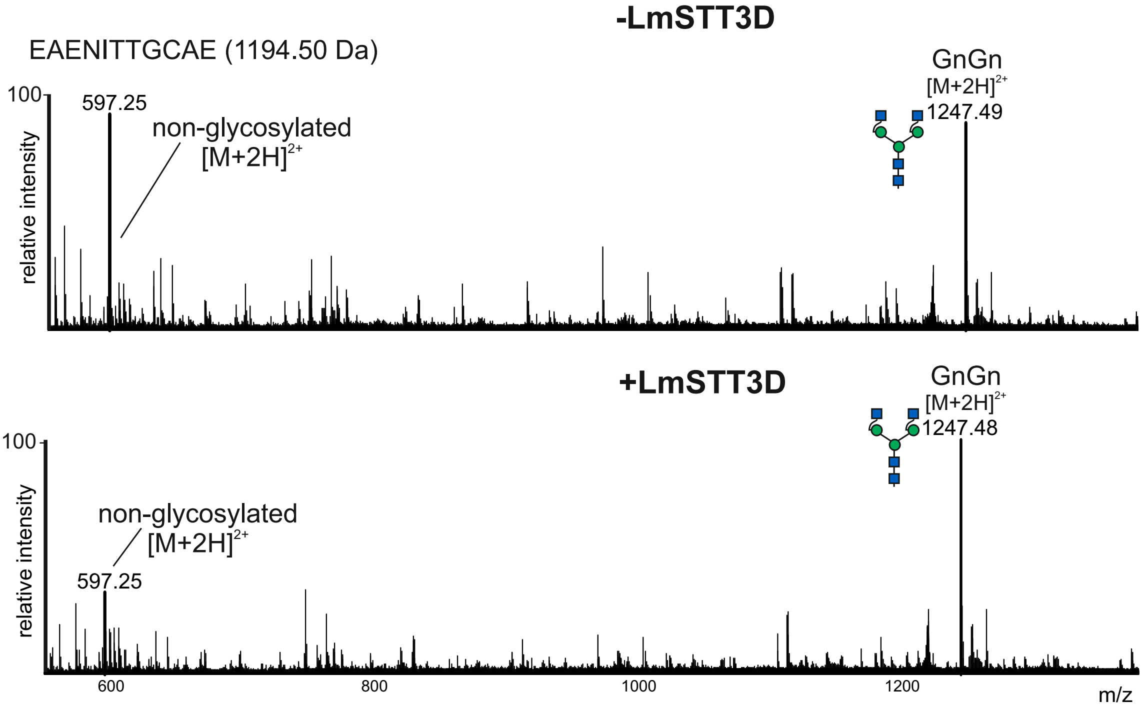

Figure S4 Mass-spectra of EPO-Fc (glyco)peptides. EPO-Fc was transiently expressed in *N. benthamiana* ΔXT/FT in the presence or absence of LmSTT3D-GFP. Purified EPO-Fc was trypsin+GluC digested and analysed by LC-ESI-MS. The mass range displaying the major glycoform from the peptide corresponding to glycosylation site 1 (due to the iodoacetamide treatment the cysteine is present as carbamidomethyl-cysteine) is shown.

(a)

IFN-γ-HA

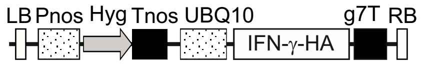

(b)

MANKHLSLSLFLVLLGLSASLAQDPYVKEAENLKKYFNAGHSDVADNGTLFLGILKNWKEESDRKIMQ
SQIVSFYFKLFKNFKDDQSIQKSVETIKEDMNVKFFNSNKKKRDDFEKLTNYSVTDLNVQRKAIHELI
QVMAELSPAAKTGKRKRSQMLFRGRRASQGSYPYDVPDYASLYPYDVPDYASLYPYDVPDYASL

Figure S5 Schematic illustration of the IFN-γ–HA expression construct (a) and the IFN-γ–HA amino acid sequence (b). (a) LB: left border; Pnos: nopaline synthase gene promoter; Hyg: hygromycin B phosphotransferase gene; Tnos: nopaline synthase gene terminator; UBQ10: *A. thaliana* ubiquitin-10 promoter; IFN-γ-HA: coding sequence of human interferon γ fused to a 3x hemagglutinin (HA) tag; g7T: agrobacterium gene 7 terminator; RB: right border. (b) Amino acid sequence of IFN-γ–HA. The signal peptide from barely alpha-amylase is marked in green. The two N-glycosylation sites (NGT and NYS) are marked in blue. A short dipeptide linker is marked in red and the 3x HA tag is highlighted in grey.
