# Supplementary material for: An oligosaccharyltransferase from Leishmania major increases the N‐glycan occupancy on recombinant glycoproteins produced in Nicotiana benthamiana
Source: Plant Biotechnol J. 2018 Mar 25;16(10):1700–9. doi: 10.1111/pbi.12906 (PMC6131413; doi:10.1111/pbi.12906)
Supplement: Supplementary file 2 — Table S1 Comparison of the N‐glycosylation site occupancy of native and recombinant glycoproteins. [file PBI-16-1700-s002.pdf]

Table S1 Comparison of the N-glycosylation site occupancy of native and recombinant glycoproteins.

Protein	N-glycosylation site (GS)	source	% glycosylated	Reference
Fc	GS1 NST	HEK293	> 90	Jez et al. 2012 and unplublished
IgG	GS1 NST	CHO	99	Rustandi et al. 2008
	GS1 NST	human serum	~100	Karnoup et al. 2007
IgE	GS3 NKT	HEK293	80	Montero-Morales et al. 2017
	GS5 NLT	HEK293	91	
	GS6 NHS	HEK293	0	
	GS3 NKT	human serum	75	Montero-Morales et al. 2017
	GS5 NLT	human serum	85	
	GS6 NHS	human serum	0	
	GS3 NKT	human serum	80	Plomp et al. 2014
	GS5 NLT	human serum	98	
IgA1	GS1 NLT	HEK293	99	Göritzer et al. 2017
	GS2 NVS	HEK293	64	
	GS1 NLT	human colostrum	< 50	Huang et al. 2015
	GS2 NVS	human colostrum	< 10	
	GS1 NLT	human serum	85	Hülsmeier et al. 2016
EPO-Fc	all sites	CHO	100	Taschwer et al. 2012
EPO	all sites	CHO	100	Gong et al. 2013
	all sites	*P. pastoris*	100	
	all sites	human serum	~100	Skibeli et al 2001
IFN-γ	GS1 + GS2	CHO	65	Wong et al. 2010
	GS1 + GS2	human blood cells	~67	Rinderknecht et al. 1984

Please note for some glycoproteins no precise data for site-specific N-glycosylation site occupancy could be obtained from literature and for mammalian cells glycosylation efficiency is dependent on culture conditions.

References

Gong, B., Burnina, I., Stadheim, T.A. and Li, H. (2013) Glycosylation characterization of recombinant human erythropoietin produced in glycoengineered Pichia pastoris by mass spectrometry. J Mass Spectrom **48**, 1308-1317.

Göritzer, K., Maresch, D., Altmann, F., Obinger, C. and Strasser, R. (2017) Exploring Site-Specific N-Glycosylation of HEK293 and Plant-Produced Human IgA Isotypes. *J Proteome Res* **16**, 2560-2570.

Huang, J., Guerrero, A., Parker, E., Strum, J.S., Smilowitz, J.T., German, J.B. and Lebrilla, C.B. (2015) Site-specific glycosylation of secretory immunoglobulin A from human colostrum. J Proteome Res **14**, 1335-1349.

Hülsmeier, A.J., Tobler, M., Burda, P. and Hennet, T. (2016) Glycosylation site occupancy in health, congenital disorder of glycosylation and fatty liver disease. Sci Rep **6**, 33927.

Jez, J., Antes, B., Castilho, A., Kainer, M., Wiederkum, S., Grass, J., Rüker, F., Woisetschläger, M. and Steinkellner, H. (2012) Significant impact of single N-glycan residues on the biological activity of Fc-based antibody-like fragments. J Biol Chem **287**, 24313-24319.

Karnoup, A.S., Kuppannan, K. and Young, S.A. (2007) A novel HPLC-UV-MS method for quantitative analysis of protein glycosylation. J Chromatogr B Analyt Technol Biomed Life Sci **859**, 178-191.

Montero-Morales, L., Maresch, D., Castilho, A., Turupcu, A., Ilieva, K.M., Crescioli, S., Karagiannis, S.N., Lupinek, C., Oostenbrink, C., Altmann, F. and Steinkellner, H. (2017) Recombinant plant-derived human IgE glycoproteomics. *J Proteomics* **161**, 81-87.

Plomp, R., Hensbergen, P.J., Rombouts, Y., Zauner, G., Dragan, I., Koeleman, C.A., Deelder, A.M. and Wuhrer, M. (2014) Site-specific N-glycosylation analysis of human immunoglobulin E. J Proteome Res 13, 536-546.

Rinderknecht, E., O'Connor, B.H. and Rodriguez, H. (1984) Natural human interferon-gamma. Complete amino acid sequence and determination of sites of glycosylation. J Biol Chem **259**, 6790-6797.

Rustandi, R.R., Washabaugh, M.W. and Wang, Y. (2008) Applications of CE SDS gel in development of biopharmaceutical antibody-based products. Electrophoresis **29**, 3612-3620.

Skibeli, V., Nissen-Lie, G. and Torjesen, P. (2001) Sugar profiling proves that human serum erythropoietin differs from recombinant human erythropoietin. Blood **98**, 3626-3634.

Taschwer, M., Hackl, M., Hernández Bort, J.A., Leitner, C., Kumar, N., Puc, U., Grass, J., Papst, M., Kunert, R., Altmann, F. and Borth, N. (2012) Growth, productivity and protein glycosylation in a CHO EpoFc producer cell line adapted to glutamine-free growth. J Biotechnol **157**, 295-303.

Wong, D.C., Wong, N.S., Goh, J.S., May, L.M. and Yap, M.G. (2010) Profiling of N-glycosylation gene expression in CHO cell fed-batch cultures. *Biotechnol Bioeng* **107**, 516-528.
